# Community‐Centric Malaria Prevention in Sub‐Saharan Africa: A Narrative Review of Pathways to Elimination and Global Health Impact

**DOI:** 10.1002/hsr2.73317

**Published:** 2026-09-28

**Authors:** Emmanuel Ifeanyi Obeagu

**Affiliations:** ^1^ Division of Hematology, Department of Biomedical and Laboratory Science Africa University Mutare Zimbabwe; ^2^ Faculty of Health Sciences, Department of Molecular Medicine and Hematology University of the Witwatersrand Johannesburg South Africa; ^3^ Department of Hematology and Immunohematology Bahir Dar University Bahir Dar Ethiopia

**Keywords:** community‐based approaches, health education, malaria prevention, Sub‐Saharan Africa, vector control

## Abstract

**Background and Aims:**

Malaria remains a major global health challenge, with the World Health Organization (WHO) African Region accounting for the overwhelming majority of malaria cases and deaths worldwide. This narrative review examines the contribution of community‐centric malaria prevention approaches to malaria elimination pathways and their broader global health implications in Sub‐Saharan Africa.

**Methods:**

A narrative literature review was conducted using PubMed/MEDLINE, Scopus, Web of Science, Google Scholar, and WHO databases. Literature published between January 2000 and December 2025 was identified using combinations of keywords including “malaria elimination,” “community engagement,” “community health workers,” “vector control,” “malaria prevention,” and “Sub‐Saharan Africa.” Eligible evidence included peer‐reviewed studies, implementation research, policy documents, and global malaria reports addressing community‐based prevention, surveillance, diagnosis, treatment, and elimination strategies. Findings were synthesized thematically.

**Results:**

The review identified community health workers, community‐directed vector control, health education, environmental management, ITN promotion, IRS participation, SMC delivery, IPTp uptake, and digital surveillance as key drivers of malaria prevention. Community‐centered approaches improved intervention acceptance, early diagnosis, treatment access, surveillance capacity, and local ownership of malaria programs. Emerging technologies, including artificial intelligence, geospatial mapping, and genomic surveillance, provide opportunities for precision malaria control when integrated with community systems.

**Conclusion:**

Community‐centered malaria prevention represents a critical pathway toward sustainable elimination in Sub‐Saharan Africa. Strengthening community empowerment, integrating scientific innovations with local knowledge, and improving health‐system partnerships are essential for accelerating malaria elimination and advancing global health equity.

## 1 Introduction

Malaria remains one of the world's most significant infectious diseases and continues to impose an enormous public health, social, and economic burden, particularly in Sub‐Saharan Africa (SSA). According to the World Health Organization (WHO) World Malaria Report 2025, the WHO African Region accounted for approximately 94% of global malaria cases and 95% of malaria‐related deaths, with children under 5 years of age and pregnant women bearing the greatest burden of morbidity and mortality. Although substantial progress has been achieved over the past two decades through the widespread implementation of insecticide‐treated nets (ITNs), indoor residual spraying (IRS), rapid diagnostic tests (RDTs), artemisinin‐based combination therapies (ACTs), seasonal malaria chemoprevention (SMC), and intermittent preventive treatment during pregnancy (IPTp), progress toward malaria elimination has slowed in many endemic countries due to persistent transmission, insecticide and antimalarial drug resistance, climate variability, population mobility, health‐system constraints, and socioeconomic inequalities [1, 2, 3]. Malaria transmission in SSA is shaped by a complex interplay of biological, environmental, behavioral, and social determinants. The distribution and abundance of *Anopheles* mosquito vectors are influenced by climatic conditions, land‐use changes, urbanization, and environmental management practices, while human behaviors such as consistent ITN use, prompt healthcare seeking, treatment adherence, and participation in vector‐control activities substantially affect transmission dynamics. Consequently, malaria elimination requires more than biomedical interventions alone; it demands sustained community engagement, multisectoral collaboration, and resilient health systems capable of adapting interventions to local epidemiological and sociocultural contexts [4, 5].

Community‐centered malaria prevention has emerged as a cornerstone of contemporary malaria elimination strategies. Unlike traditional top‐down approaches that primarily focus on delivering interventions to communities, community‐centered models actively involve local populations in planning, implementing, monitoring, and evaluating malaria control activities. Community health workers (CHWs), village health committees, local leaders, women's groups, schools, and civil society organizations contribute to health education, environmental sanitation, vector surveillance, early diagnosis, treatment adherence, distribution of preventive commodities, and reporting of malaria cases. Such participatory approaches improve intervention coverage, increase public trust, strengthen local ownership, and enhance the sustainability of malaria programs [6, 7, 8]. Recent advances in digital health, geographic information systems (GIS), artificial intelligence (AI), genomic surveillance, and precision public health have further expanded opportunities for integrating community participation with data‐driven malaria control. Mobile health platforms facilitate real‐time disease reporting and community surveillance, while AI‐assisted predictive modeling and geospatial mapping support targeted deployment of vector‐control interventions and early outbreak detection. Similarly, molecular surveillance of parasites and vectors can inform resistance monitoring and guide evidence‐based public health responses. Integrating these technological innovations with community‐based programs offers promising opportunities to improve the efficiency and effectiveness of malaria elimination efforts across diverse transmission settings [9, 10, 11].

Although several systematic reviews have evaluated specific community‐based malaria interventions, including community health workers, insecticide‐treated net utilization, or health education programs, these reviews generally focus on individual interventions or narrowly defined outcomes. Few have comprehensively synthesized the broader implementation pathways through which community engagement, health‐system strengthening, behavioral interventions, digital technologies, emerging vector‐control innovations, and policy frameworks collectively contribute to malaria elimination in Sub‐Saharan Africa (Figure 1). A narrative review is therefore appropriate because it enables integration of evidence from diverse study designs, implementation research, policy documents, WHO reports, and operational experiences, providing a broader conceptual understanding of community‐centered malaria prevention within the evolving elimination landscape [12, 13]. Accordingly, this narrative review synthesizes current evidence on community‐centric malaria prevention strategies in Sub‐Saharan Africa, critically examines their effectiveness, implementation challenges, and opportunities for strengthening elimination programs, and explores how integrating community empowerment with scientific innovation, digital technologies, and health‐system partnerships can accelerate malaria elimination and contribute to broader global health goals, including health equity, universal health coverage, and sustainable development.

**Figure 1:**
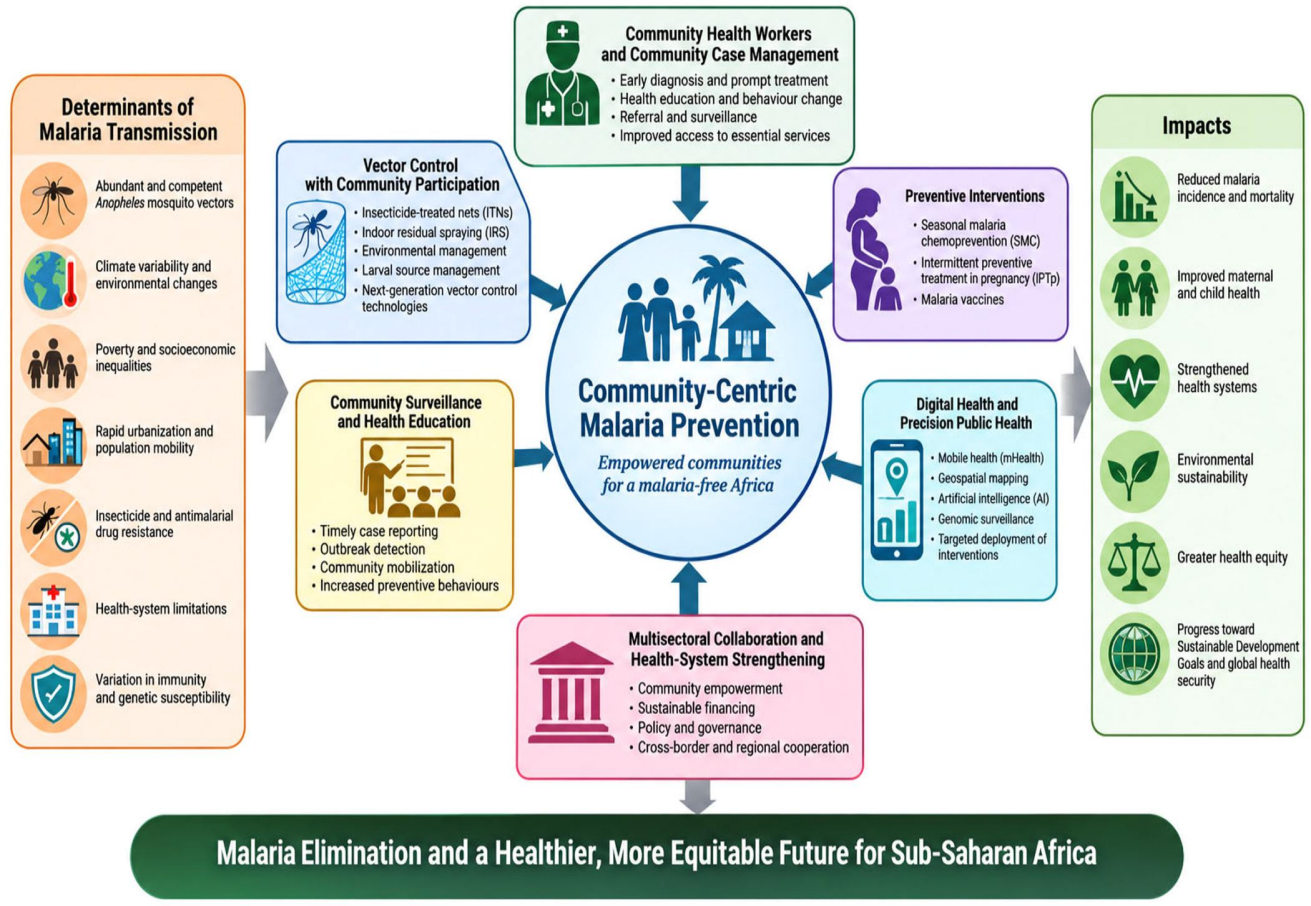
Conceptual framework for community‐centric malaria prevention in Sub‐Saharan Africa: Integrated strategies for malaria elimination and global health impact.

### 1.1 Aim

The aim of this narrative review is to critically examine and synthesize current evidence on community‐based approaches to malaria prevention in Sub‐Saharan Africa.

## 2 Methods

### 2.1 Review Design and Approach

This article was developed as a narrative review examining community‐centric approaches to malaria prevention and their contribution to malaria control, elimination, health equity, and broader global health outcomes in sub‐Saharan Africa. The review was designed to provide a critical and integrative synthesis rather than a systematic review or meta‐analysis. The methodological quality and completeness of the narrative review were guided by the Scale for the Assessment of Narrative Review Articles (SANRA) proposed by Baethge et al. [14], with particular attention to appropriate justification of the review's importance, clear explanation of the literature search strategy, adequate referencing, scientific reasoning, and presentation of relevant data.

### 2.2 Literature Identification

Relevant literature was identified through searches of major biomedical and multidisciplinary databases, including PubMed/MEDLINE, Scopus and Web of Science and ScienceDirect. Additional relevant publications were identified through reference‐list screening of key articles and authoritative reports from organizations including the World Health Organization, World Health Organization Regional Office for Africa, UNICEF, and national malaria‐control programs. Search concepts combined terms related to malaria, community participation, prevention, elimination, vector control, surveillance, health workers, chemoprevention, vaccination, digital health, and sub‐Saharan Africa. Representative search terms included: *malaria*, *malaria prevention*, *malaria elimination*, *community engagement*, *community participation*, *community health workers*, *integrated vector management*, *insecticide‐treated nets*, *indoor residual spraying*, *seasonal malaria chemoprevention*, *intermittent preventive treatment*, *malaria vaccine*, *malaria surveillance*, *mobile health*, *digital surveillance*, *climate change*, *health equity*, and *sub‐Saharan Africa*.

### 2.3 Eligibility and Selection of Evidence

Priority was given to peer‐reviewed studies, systematic reviews, meta‐analyzes, scoping reviews, implementation studies, epidemiological investigations, community‐based interventions, and authoritative technical reports that provided substantive evidence relevant to malaria prevention or elimination in sub‐Saharan Africa. Literature addressing community engagement, integrated vector management, community health‐worker delivery, maternal and child malaria prevention, surveillance, digital technologies, vaccination, environmental management, climate‐related malaria risks, and health equity was particularly emphasized. Studies from outside sub‐Saharan Africa were considered selectively when they provided important conceptual, technological, or implementation evidence directly applicable to malaria elimination in African settings. No formal quantitative pooling of effect estimates was undertaken.

### 2.4 Narrative Synthesis

Evidence was critically organized into interconnected thematic domains: (1) community engagement and ownership; (2) community‐based vector control; (3) community health workers; (4) chemoprevention and maternal malaria prevention; (5) malaria vaccination; (6) community‐based surveillance and digital health; (7) climate‐resilient malaria prevention; (8) social and structural determinants; (9) mobile and cross‐border populations; (10) insecticide and antimalarial resistance; and (11) health equity and pathways toward elimination. Particular attention was given to how community participation can influence intervention coverage, acceptability, adherence, early diagnosis, treatment seeking, surveillance sensitivity, environmental management, and sustainability. Evidence from recent studies on mobile application‐based malaria surveillance and vector‐control integration, malaria‐control effectiveness in Nigeria, community‐based IPTp strategies in sub‐Saharan Africa, and stakeholder participation in integrated vector management in western Kenya was incorporated to strengthen the contemporary and community‐focused perspective of the review.

### 2.5 Quality and Methodological Considerations

The review was assessed using the SANRA framework, which is appropriate for narrative reviews and emphasizes justification of the review, description of the literature search, referencing, evidence of scientific reasoning, and appropriate presentation of data. The review does not claim to be a systematic review, and PRISMA was therefore not used as the principal reporting framework. The synthesis was interpretive and thematic, with emphasis on integrating epidemiological evidence, implementation experience, community perspectives, health‐system considerations, and emerging technologies.

### 2.6 Limitations of the Review Method

As a narrative review, the approach does not provide the exhaustive study identification, formal risk‐of‐bias assessment, or quantitative pooled estimates characteristic of systematic reviews and meta‐analyzes. Selection and interpretation of literature may therefore be influenced by author judgment. Nevertheless, the structured search strategy, explicit thematic organization, use of diverse evidence sources, and application of SANRA were intended to enhance transparency, scientific rigor, and reproducibility of the narrative synthesis.

### 2.7 Community Health Workers and Malaria Prevention

Community health workers (CHWs) have become a cornerstone of malaria prevention and control efforts across sub‐Saharan Africa, particularly in settings where access to formal healthcare services remains limited. Positioned within the communities they serve, CHWs operate at the interface between households and health systems, enabling timely, culturally appropriate, and cost‐effective delivery of malaria interventions. Their proximity to the population fosters trust, enhances communication, and promotes sustained engagement with preventive measures [14]. In the context of malaria prevention, CHWs play a multifaceted role that extends beyond basic service delivery. They are actively involved in the distribution and promotion of insecticide‐treated nets (ITNs), ensuring not only equitable access but also correct and consistent usage. Through household visits and community sensitization campaigns, CHWs address misconceptions, reinforce behavioral change, and encourage early care‐seeking practices. Their involvement has been shown to significantly improve adherence to preventive strategies, particularly among vulnerable groups such as pregnant women and young children [15].

CHWs are also central to integrated community case management (iCCM), where they provide rapid diagnostic testing and administer appropriate antimalarial treatment. This decentralized approach reduces delays in diagnosis and treatment, which is critical in preventing disease progression and transmission. In addition, CHWs support the implementation of seasonal malaria chemoprevention (SMC) programs, delivering prophylactic medications to at‐risk pediatric populations in highly endemic regions. Their familiarity with local demographics ensures efficient targeting and high coverage rates [16]. Beyond direct clinical and preventive functions, CHWs contribute to community‐based surveillance systems. By documenting malaria cases and reporting trends through structured tools—often supported by mobile health technologies—they facilitate real‐time data collection that informs public health decision‐making. This localized surveillance capacity strengthens early warning systems and enables more responsive, targeted interventions [17]. CHWs also play a critical role in fostering community ownership of malaria control programs. By engaging local leaders, schools, and social networks, they help embed malaria prevention within the social fabric of the community. This participatory approach enhances program acceptability and sustainability, transforming malaria control from an externally driven initiative into a shared community responsibility [18]. Inconsistent support can undermine performance and retention, highlighting the need for stronger integration of CHWs into national health systems. Investments in capacity building, digital tools, and supportive supervision are essential to maximize their potential [19].

### 2.8 Relationship Between Community Health Workers and Emerging Vector Control Technologies

CHWs serve as a critical interface between health systems and communities, playing an increasingly important role in the successful implementation, acceptance, and monitoring of emerging vector control technologies. While the development of innovative interventions such as dual‐active ingredient insecticide‐treated nets, next‐generation IRS formulations, attractive targeted sugar baits, spatial repellents, larval source management, SIT, and genetically modified mosquito technologies has expanded the malaria control toolkit, their effectiveness depends largely on community awareness, acceptance, and sustained utilization. CHWs facilitate these processes by delivering culturally appropriate health education, addressing misconceptions, engaging community leaders, and promoting informed participation in vector control programs [15]. Beyond community sensitization, CHWs contribute to the operational implementation and evaluation of new vector control strategies. They assist in identifying high‐risk households, supporting the distribution and correct use of next‐generation insecticide‐treated nets, mobilizing communities during IRS campaigns, monitoring intervention coverage, and encouraging adherence to recommended preventive practices. Their routine household visits also enable early identification of operational challenges, such as improper net usage, reduced acceptance of IRS, or environmental conditions that favor mosquito breeding, allowing timely corrective actions by malaria control programs [16].

CHWs increasingly contribute to surveillance systems with digital health technologies, including mobile applications for reporting malaria cases, documenting vector control coverage, mapping breeding sites, and identifying potential insecticide resistance hotspots. These community‐generated data strengthen national malaria surveillance systems by enabling real‐time monitoring of intervention performance and facilitating targeted deployment of resources. In addition, CHWs can support community‐based entomological surveillance by reporting changes in mosquito density, biting behavior, or local environmental conditions that may influence transmission dynamics [17]. The successful introduction of advanced technologies such as gene‐drive mosquitoes, Wolbachia‐based vector control, and precision vector surveillance will also require sustained community engagement, ethical communication, and public trust. CHWs are well positioned to facilitate dialog between researchers, public health authorities, and local communities by explaining the scientific rationale, potential benefits, possible risks, and regulatory safeguards associated with these innovations. Their involvement promotes informed community participation and helps address concerns that may otherwise limit implementation [18].

### 2.9 Health Education and Behavior Change Communication

Health education and behavior change communication (BCC) are vital components of community‐based malaria prevention, aiming to enhance awareness, knowledge, and adoption of protective behaviors among populations at risk. Effective communication strategies help bridge the gap between scientific knowledge and local practices by addressing misconceptions, cultural beliefs, and social norms that may hinder the consistent use of malaria prevention tools such as insecticide‐treated nets (ITNs) and intermittent preventive treatment in pregnancy (IPTp). In Sub‐Saharan Africa, tailored BCC interventions have been instrumental in fostering sustainable behavior change and improving community participation in malaria control efforts [19].

BCC interventions utilize diverse platforms and methods to disseminate malaria‐related information, including interpersonal counseling by community health workers, community meetings, school programs, local media campaigns, and innovative tools such as drama, songs, and storytelling. These culturally sensitive approaches resonate with diverse audiences, including those with low literacy levels, and encourage active engagement. For instance, community drama performances have effectively conveyed malaria prevention messages, fostering understanding and motivating behavior change in rural settings. Moreover, school‐based health education empowers children and adolescents to become advocates for malaria prevention within their families and communities [19]. Sustained behavior change requires repeated, clear, and context‐specific messaging that aligns with local values and addresses barriers to intervention uptake. Collaborative involvement of community leaders, religious figures, and other influential stakeholders enhances message credibility and reach. However, challenges remain in ensuring the consistency, quality, and evaluation of BCC programs, particularly in resource‐constrained settings. Future efforts should focus on integrating BCC with broader health promotion initiatives, leveraging mobile technologies for real‐time communication, and developing evidence‐based strategies to monitor and reinforce positive behaviors over time (Table 1) [26, 27].

**Table 1:** Health education and behavior change communication (BCC) in malaria prevention.

Health education/BCC intervention	Target audience	Expected outcome	Evidence source(s)
Community malaria education and dialog	Households and community leaders	Improved malaria knowledge and preventive practices	[20]
Promotion of consistent ITN use	Households, caregivers, children	Increased ITN utilization	[21]
Community mobilization for vector control	Households and local leaders	Improved acceptance and coverage of vector‐control interventions	[22]
BCC on early care‐seeking and treatment	Caregivers and adults	Reduced delays in diagnosis and treatment	[23]
Community engagement in integrated vector management	Communities, local leaders, stakeholders	Increased community ownership and sustainability	[24]
Maternal malaria‐prevention education	Pregnant women and families	Improved uptake of IPTp and antenatal malaria‐prevention services	[25]
Community communication concerning malaria vaccination	Parents, caregivers, communities	Improved vaccine awareness, confidence, and uptake	[25]

### 2.10 Participatory and Integrated Community‐Based Interventions

Participatory and integrated community‐based interventions represent a holistic approach to malaria prevention that actively involves communities in decision‐making, planning, and implementation processes. Unlike top‐down models, participatory methods empower local populations to identify their specific needs, leverage indigenous knowledge, and mobilize collective action against malaria. This approach fosters a sense of ownership and responsibility, which is critical for sustaining preventive behaviors and environmental management practices over the long term [8, 28]. Integrated community‐based interventions combine malaria prevention with other health and social programs, such as maternal and child health, nutrition, water, sanitation, and hygiene (WASH), and HIV/AIDS services. By addressing overlapping determinants of health and leveraging shared resources, integrated programs enhance efficiency, reduce duplication, and improve overall health outcomes. For example, combining malaria education and ITN distribution with antenatal care visits ensures pregnant women receive comprehensive care, including intermittent preventive treatment in pregnancy (IPTp), nutritional counseling, and malaria prevention education in one accessible setting [29, 30].

Successful participatory interventions often include community‐led environmental management activities, such as clearing mosquito breeding sites, improving drainage, and promoting waste management to reduce vector populations. The establishment of community health committees and malaria action groups facilitates sustained engagement, monitoring, and accountability. Additionally, participatory mapping and local surveillance systems help tailor interventions to specific geographic and epidemiological contexts. Despite these benefits, challenges such as limited funding, variable community motivation, and coordination complexities can impede implementation. Strengthening partnerships among communities, health systems, and non‐governmental organizations is essential for scaling and sustaining these integrated approaches (Table 2) [33, 34].

**Table 2:** Participatory and integrated community‐based interventions for malaria prevention.

Participatory/integrated intervention	Community participants/target population	Principal malaria‐prevention function	Evidence source(s)
Community‐led integrated vector management	Households, community leaders, environmental stakeholders	Local vector surveillance, environmental management and coordinated vector control	[20]
Community health‐worker malaria services	Rural and hard‐to‐reach populations	Community diagnosis, treatment, referral, education and surveillance	[20]
Community‐based IPTp delivery	Pregnant women and communities	Increased access to and uptake of preventive treatment	[21]
ITN distribution and community promotion	Households, children and pregnant women	Reduction of human–mosquito contact	[21]
Community‐supported IRS	Households and local leaders	Reduction of indoor vector populations	[22]
Community environmental management	Households, local authorities and community groups	Reduction of productive mosquito breeding sites	[31]
Community malaria surveillance	CHWs, community volunteers and health facilities	Early detection, reporting and response to residual transmission	[32]
Mobile/digital community surveillance	CHWs, surveillance teams and communities	Real‐time reporting, geospatial targeting and integrated response	[31]
Malaria vaccination integrated with community prevention	Children, caregivers and communities	Reduction of malaria morbidity and mortality alongside existing interventions	[22]
Cross‐sectoral community participation	Health, education, environmental and local‐government stakeholders	Sustainable, locally adapted malaria elimination	[21]

### 2.11 Emerging Drug Resistance and Alternative Therapies

The escalating emergence of resistance to frontline antimalarial drugs poses a significant threat to malaria control in Sub‐Saharan Africa. Artemisinin‐based combination therapies (ACTs), which have been the cornerstone of treatment, are increasingly compromised by parasite mutations leading to delayed clearance times and treatment failures. Surveillance studies have detected resistance markers spreading beyond Southeast Asia into parts of Africa, raising urgent concerns about the durability of current regimens. This scenario underscores the need for robust community‐level surveillance systems that enable early detection of resistance patterns [35, 36]. In response, alternative therapeutic approaches are being explored. Triple ACTs, combining three drugs with distinct mechanisms, show promise in overcoming resistance and are under field evaluation. New compounds such as tafenoquine, a single‐dose antimalarial with long half‐life, offer convenience and improved compliance, while ferroquine exhibits potent activity against resistant strains. Traditional medicine, often underutilized, is also being reexamined; several plant‐derived compounds exhibit antimalarial properties and could complement pharmacological therapies if rigorously validated [37, 38]. Community health workers (CHWs) play a pivotal role in implementing updated treatment protocols, recognizing signs of treatment failure, and facilitating referrals. Ensuring continuous training and adequate supply chains for novel therapies at the community level is critical. Additionally, public awareness campaigns addressing drug resistance can promote adherence to prescribed treatments, reducing the risk of suboptimal dosing that fuels resistance development. Integration of pharmacovigilance with community‐based programs will strengthen early identification and containment of resistance hotspots [39, 40].

### 2.12 Advances in Vector Control Technologies

Vector control remains a cornerstone of malaria prevention, and recent technological advances have broadened the arsenal available to communities. Next‐generation insecticide‐treated nets (ITNs) now incorporate multiple active ingredients, such as pyrethroids combined with piperonyl butoxide, which inhibit mosquito detoxification enzymes and help overcome insecticide resistance. Spatial repellents, which create protective “zones” without requiring direct contact, are gaining traction as complementary tools in peri‐domestic settings [40, 41]. Innovative strategies such as Attractive Toxic Sugar Baits (ATSB) exploit mosquitoes' sugar feeding behavior to deliver insecticides in a targeted manner, reducing mosquito populations with minimal environmental impact. Endectocides, such as ivermectin, administered to humans or livestock, kill mosquitoes that feed on treated hosts, offering a systemic vector control method [42].

Genetic vector control, particularly gene drive technologies, is a revolutionary approach aiming to reduce mosquito fertility or modify vector competence. Field trials in controlled settings are underway, though widespread deployment requires extensive community engagement to address ethical, ecological, and social concerns [43]. Furthermore, drone technology is being leveraged for larval source management, enabling rapid identification and treatment of breeding sites in inaccessible areas. These innovations, when integrated into community‐based programs, can enhance vector suppression effectiveness, reduce malaria transmission, and delay insecticide resistance [44].

### 2.13 Digital Health and Mobile Technology

The rise of mobile and digital technologies presents unprecedented opportunities to strengthen community‐based malaria prevention in Sub‐Saharan Africa. Mobile health (mHealth) platforms enable real‐time data collection, case reporting, and supply chain management, addressing critical gaps in malaria program monitoring and responsiveness. CHWs equipped with smartphones or tablets can capture diagnostic and treatment data, enabling health systems to track trends, detect outbreaks, and allocate resources efficiently [45]. Health education delivered via SMS, interactive voice response, and social media campaigns enhances community awareness and encourages preventive behaviors such as consistent use of ITNs and early treatment seeking. Tailoring content in local languages and using culturally relevant messaging increases engagement and impact [45].

AI and machine learning models are being piloted to predict malaria outbreaks based on climate, environmental, and epidemiological data. These predictive tools facilitate timely community mobilization and pre‐emptive vector control activities. Mobile apps also enable communities to report vector breeding sites or medication stock‐outs, fostering transparency and accountability [46, 47]. Challenges remain, including limited digital literacy, network coverage gaps, and data privacy concerns, particularly in rural areas. Partnerships between governments, NGOs, and telecom providers are vital to expanding access and developing user‐friendly platforms tailored to local contexts [48].

### 2.14 Immunological and Genetic Factors Influencing Malaria Susceptibility: Implications for Community‐Centered Prevention

Host immunological and genetic factors play an important role in determining susceptibility to malaria infection, disease severity, and clinical outcomes. Protective immunity against *Plasmodium falciparum* develops gradually following repeated exposure and involves coordinated innate and adaptive immune responses, including macrophages, dendritic cells, natural killer cells, CD4^+^ and CD8^+^ T lymphocytes, and parasite‐specific antibodies. However, immunity is often incomplete and wanes in areas where transmission declines, leaving young children, pregnant women, migrants, and immunologically naïve populations particularly vulnerable to infection. Variations in cytokine responses, inflammatory regulation, and antibody‐mediated immunity further contribute to differences in disease susceptibility and treatment outcomes among individuals and communities. Several inherited genetic traits common in malaria‐endemic regions influence susceptibility to infection and disease severity. Classical examples include the sickle cell trait (HbAS), α‐thalassemia, glucose‐6‐phosphate dehydrogenase (G6PD) deficiency, Southeast Asian ovalocytosis, and Duffy antigen polymorphisms, which confer varying degrees of protection against severe malaria or specific *Plasmodium* species. In addition, polymorphisms affecting immune‐regulatory genes, human leukocyte antigen (HLA) molecules, Toll‐like receptors (TLRs), cytokines, and complement pathways have been associated with differences in host immune responses and clinical manifestations. At the parasite level, genetic mutations associated with antimalarial drug resistance and increasing insecticide resistance among *Anopheles* mosquitoes continue to threaten malaria control efforts and underscore the need for ongoing molecular surveillance [49, 50].

Although these immunological and genetic advances are primarily biomedical, they have important implications for community‐centered malaria prevention. CHWs can play a pivotal role in translating complex scientific knowledge into practical public health interventions by educating communities about populations at increased risk, promoting early diagnosis and treatment, supporting adherence to preventive measures such asITNs, sSMC, and IPTp, and facilitating timely referral of high‐risk individuals. Community engagement is also essential for improving awareness and acceptance of malaria vaccines, particularly the WHO‐recommended RTS,S/AS01 and R21/Matrix‐M vaccines, whose implementation requires high vaccination coverage, effective communication, and community trust [51]. Furthermore, advances in parasite and vector genomics are increasingly supporting community‐level malaria surveillance. Molecular surveillance can identify transmission hotspots, monitor emerging antimalarial drug and insecticide resistance, and guide the targeted deployment of interventions. When integrated with community‐based surveillance systems, digital reporting platforms, and GIS, genomic information can support precision public health approaches that allocate resources more efficiently according to local transmission dynamics. Community participation in surveillance activities enhances the timeliness and completeness of reporting, strengthening the responsiveness of malaria control programs [52].

## 3 Results

The structured literature search identified studies and reports examining community‐centered malaria prevention and elimination strategies implemented across Sub‐Saharan Africa. Following screening of titles, abstracts, and full‐text articles according to the predefined eligibility criteria, the final evidence base comprised peer‐reviewed original studies, systematic and narrative reviews, implementation and operational research, WHO technical reports, and policy documents. The included evidence was synthesized thematically according to the major domains of community participation and their contribution to malaria prevention and elimination (Figure 1).

### 3.1 Community Health Workers and Community‐Based Case Management

The largest body of evidence evaluated the role of CHWs in malaria prevention and control. Studies consistently demonstrated that CHWs improve access to RDTs, facilitate prompt initiation of ACT, promote treatment adherence, provide health education, and strengthen referral systems, particularly in rural and underserved communities. Community‐based case management was associated with earlier diagnosis, reduced treatment delays, improved healthcare‐seeking behavior, and lower malaria‐related morbidity among children under 5 years of age and pregnant women.

### 3.2 Community Participation in Vector Control

Numerous studies reported that active community engagement substantially improves the effectiveness of vector control interventions. Community‐led promotion and consistent utilization of ITN increased household ownership and appropriate net use, while participation in IRS campaigns improved household acceptance and intervention coverage. Community‐driven environmental sanitation, including drainage of stagnant water, removal of mosquito breeding sites, waste management, and improved sanitation practices, further contributed to reducing vector density and malaria transmission. These interventions were most successful when implemented alongside culturally appropriate health education and community mobilization.

### 3.3 Seasonal Malaria Chemoprevention and Preventive Interventions

Evidence from implementation studies demonstrated that community‐based delivery of SMC significantly improved treatment coverage among eligible children in highly seasonal transmission settings. Community involvement enhanced caregiver acceptance, adherence to treatment schedules, and timely administration of preventive medicines. Similarly, community education programs increased the uptake of IPTp by promoting early antenatal care attendance, improving awareness of malaria risks during pregnancy, and supporting treatment adherence.

### 3.4 Community Surveillance and Health Education

Community participation strengthened malaria surveillance through timely reporting of suspected cases, household monitoring, and collaboration with local health facilities. CHWs and community volunteers contributed to early outbreak detection, improved disease reporting, and enhanced monitoring of intervention coverage. Health education campaigns addressing malaria transmission, prevention practices, early healthcare seeking, and treatment adherence consistently improved community knowledge, attitudes, and preventive behaviors, thereby supporting sustained reductions in malaria transmission.

### 3.5 Digital Health and Emerging Technologies

An increasing number of studies described the application of digital health technologies to strengthen community‐based malaria programs. Mobile health (mHealth) platforms enabled real‐time reporting of malaria cases, monitoring of intervention coverage, and communication between CHWs and healthcare facilities. GIS, geospatial mapping, and AI‐based predictive models were increasingly used to identify transmission hotspots, forecast outbreaks, optimize resource allocation, and guide targeted vector control interventions. Emerging molecular and genomic surveillance approaches also demonstrated potential for monitoring parasite diversity, detecting antimalarial drug resistance, and tracking insecticide resistance in mosquito populations, thereby supporting precision malaria control.

### 3.6 Integration of Community Engagement with Scientific Innovation

The reviewed literature emphasized that the greatest impact was achieved when community participation was integrated with advances in diagnostics, vaccines, surveillance technologies, and vector control innovations. CHWs played an important role in facilitating community acceptance of next‐generation ITNs, supporting IRS campaigns, promoting malaria vaccine uptake, educating communities about emerging interventions, and contributing to digital surveillance systems. This integration strengthened program implementation while enhancing community trust, ownership, and sustainability.

## 4 Discussion

This narrative review highlights that community‐centered malaria prevention is a fundamental component of malaria elimination strategies in Sub‐Saharan Africa (SSA). The evidence synthesized demonstrates that sustained community engagement enhances the effectiveness of established malaria interventions while providing a platform for integrating emerging technologies and precision public health approaches. Rather than functioning as passive recipients of malaria control programs, communities have increasingly become active partners in vector control, disease surveillance, health promotion, early case detection, and implementation of preventive interventions. These findings are consistent with the WHO Global Technical Strategy for Malaria 2016–2030, which recognizes community participation as an essential pillar for achieving malaria elimination and sustaining elimination gains. One of the strongest findings of this review is the central role of CHWs in strengthening malaria programs. Across multiple studies, CHWs consistently improved access to rapid diagnostic testing, prompt treatment with artemisinin‐based combination therapies (ACTs), health education, referral systems, and community surveillance, particularly in rural and underserved populations. Previous systematic reviews have similarly reported that community case management significantly reduces delays in diagnosis and treatment while improving treatment adherence and reducing malaria‐related morbidity among children under 5 years of age. Beyond clinical management, this review demonstrates that CHWs also facilitate implementation of vector‐control interventions, promote uptake of IPTp and SMC, support digital surveillance, and foster community trust, thereby expanding their contribution from service delivery to implementation and program sustainability [53, 54].

The review also reinforces the importance of community participation in integrated vector management. Evidence consistently indicates that the effectiveness of ITNs, IRS, larval source management, and environmental sanitation depends not only on the availability of these interventions but also on community acceptance, appropriate utilization, and sustained behavioral change. Community‐led environmental management—including elimination of mosquito breeding sites, waste management, and improved sanitation—complements conventional vector‐control measures by addressing local ecological determinants of malaria transmission. These findings support previous reviews demonstrating that integrated vector management is most successful when technical interventions are combined with active community engagement and culturally appropriate health education. An important contribution of this review is the integration of emerging scientific and technological innovations within the framework of community‐centered malaria prevention. Digital health platforms, mobile reporting systems, GIS, AI, genomic surveillance, and precision public health approaches are increasingly enhancing malaria surveillance and resource allocation. However, the successful implementation of these innovations depends heavily on community participation and the capacity of CHWs to facilitate technology adoption, improve public understanding, address misconceptions, and ensure equitable access. Likewise, the introduction of WHO‐recommended malaria vaccines, including RTS,S/AS01 and R21/Matrix‐M, highlights the growing importance of community engagement in achieving high vaccine acceptance and coverage. These findings suggest that technological innovation and community participation should be viewed as complementary rather than independent strategies [55, 56, 57].

The review further demonstrates that immunological and genetic advances have important implications for community‐based malaria prevention. Although host genetic factors, naturally acquired immunity, and molecular surveillance are primarily biomedical concepts, they can inform targeted health education, identification of high‐risk populations, monitoring of parasite and vector resistance, and precision deployment of interventions. Integrating genomic surveillance with community‐based reporting systems may strengthen early detection of antimalarial drug resistance and insecticide resistance, thereby supporting more responsive malaria control programs. Such integration represents an emerging opportunity for precision public health in malaria‐endemic settings. This review possesses several strengths. It provides a comprehensive synthesis of diverse evidence encompassing community engagement, implementation science, policy documents, WHO guidance, technological innovations, and health‐system perspectives. By integrating biomedical, behavioral, technological, and policy dimensions, the review offers a broader conceptual framework than reviews focusing on individual malaria interventions. In addition, the structured literature search, transparent methodology, thematic evidence synthesis, and inclusion of recent WHO recommendations improve the scientific robustness and reproducibility of the review.

Nevertheless, some limitations should be acknowledged. As a narrative review, the study was not designed to provide quantitative pooled effect estimates or formal comparative analyzes between interventions. Although a structured search strategy and predefined eligibility criteria were employed, publication bias and selection bias cannot be completely excluded. Considerable heterogeneity among study designs, intervention types, outcome measures, and epidemiological settings limited direct comparisons across studies. Furthermore, most available evidence originated from high‐transmission settings, which may reduce the generalizability of findings to areas approaching malaria elimination or experiencing unstable transmission. Future systematic reviews and meta‐analyzes evaluating specific community‐centered interventions would complement the broader conceptual synthesis presented in this review. The findings have important implications for policy and future research. National malaria programs should prioritize strengthening CHW programs, expanding community‐based surveillance, integrating digital health technologies into routine malaria services, promoting community ownership of vector‐control interventions, and enhancing multisectoral collaboration. Greater investment is needed to evaluate the long‐term effectiveness, scalability, cost‐effectiveness, and sustainability of community‐led interventions, particularly in the context of climate change, emerging vector‐control technologies, malaria vaccine implementation, and precision public health. Future research should also examine strategies for integrating genomic surveillance, AI‐assisted decision support, and community engagement into routine malaria elimination programs while ensuring equitable access across diverse populations [55, 56, 57].

## 5 Conclusion

Community‐centered malaria prevention has become an indispensable pillar of malaria control and elimination efforts in Sub‐Saharan Africa, complementing biomedical interventions through sustained community engagement, local ownership, and strengthened primary healthcare systems. This review demonstrates that active participation of communities, supported by CHWs, significantly enhances the uptake and effectiveness of key interventions, including ITNs, IRS, SMC, IPTp, environmental management, early diagnosis, prompt treatment, and community‐based surveillance. These participatory approaches not only improve intervention coverage and adherence but also foster trust, resilience, and long‐term sustainability of malaria programs. The review further highlights that achieving malaria elimination requires the integration of community engagement with emerging scientific and technological innovations. Digital health platforms, AI, geospatial mapping, genomic surveillance, next‐generation vector control technologies, and malaria vaccines offer unprecedented opportunities to enhance precision public health. However, their successful implementation depends on meaningful community involvement, effective health communication, ethical governance, and strong partnerships between communities, researchers, healthcare providers, and policymakers. Community health workers remain central to this process by facilitating technology adoption, supporting surveillance, addressing misconceptions, and ensuring equitable access to preventive and diagnostic services.

## Author Contributions

**Emmanuel Ifeanyi Obeagu:** conceptualization, methodology, writing – original draft, supervision, writing – review and editing, visualization, validation.

## Funding

The author has nothing to report.

## Ethics Statement

Ethical approval was not required because this narrative review used published literature and did not involve human participants, patient‐level clinical information, or patient images.

## Consent

The author has nothing to report.

## Conflicts of Interest

The author declares no conflicts of interest. No financial relationship influenced study design; collection, analysis, or interpretation of data; writing of the report; or the decision to submit the manuscript for publication.

## Reference Integrity Statement

The reference list was checked for retractions and published corrections before resubmission. No cited article was identified as retracted. Where corrections were identified, the citation remained relevant to the statement supported.

## Transparency Statement

Emmanuel Ifeanyi Obeagu affirms that this article is an honest, accurate, and transparent account of the study being reported; that no important aspects of the study have been omitted; and that any discrepancies from the study as planned have been explained.

## Data Availability

Data sharing not applicable to this article as no datasets were generated or analyzed during the current study. No new dataset was generated or analyzed for this narrative review.
